# The Book World of Medicine and Science

**Published:** 1900-01-13

**Authors:** 


### THE BOOK WORLD OF MEDICINE AND SCIENCE.

DISEASES OF THE NERVOUS SYSTEM. By H. CAMPBELL THOMSON, M.D. (London: Baillière, Tindall, and Cox, 1899. Pp. 123. Illustrated. Price 4s.)

It is in no conventional sense that we say that Dr. Campbell Thomson's work supplies a long-felt want. Neurology—perhaps the only department of medicine that is in any sense a science—has increased so enormously of late in importance that it is an absolute necessity for the practising physician to have at his elbow some succinct presentment of the steps in diagnosis. Such a presentment, lucid and accurate, is now given to us by Dr. Campbell Thomson. It would be foolish and unnecessary to say this is a work of great originality or of illuminating hypothesis. It is a careful and logical marshalling of ascertained facts and is so admirably arranged that one almost feels the diagnosis of nerve disease—given always accurate observation—as much a logical process as the construction of a syllogism. In truth, the author owes much to the influence of Sir W. Gowers' supremely logical mind, and the student who diligently pursues the methods here set forth will be able with greater profit to read Sir W. Gowers' luminous pages. The plan of this work is simple, almost inevitable indeed; the general structure of the central nervous system being first discussed, then the sensory and motor systems, individual, and grouped muscles. Finally there are chapters on reflexes, on localisation, and on disorders of gait. Of course the new conceptions of neurology, the neuron, axon, and neuraxon, are elaborated, and references to the latest work are commendably frequent. But if we have to find a fault with Dr. Thomson we must notice that he scarcely insists on the very important fact that, in the central nervous system, movements rather than muscles are represented. And in future editions it would be well if Dr. Thomson a little enlarged the scope of his work, expanded some brevities, and repaired a few omissions.

CLINICAL STUDIES IN VICE AND INSANITY. By GEORGE R. WILSON, M.D. (Edinburgh: William F. Clay. 8vo., pp. 225. 7s. 6d.)

DR. WILSON divides his work, as Cæsar did Gaul, into three parts. The first contains 42 pages of letterpress proper, and deals with the various phenomena of drunkenness. The second part gives us the history of 12 typical cases of alcoholism, and the third is devoted almost entirely to the history of 15 cases of insanity, with various and excellent reflections on etiology, pathology, and treatment. Dr. Wilson thinks it would be advisable to group the various forms of drunkenness under one generic name. Probably he is right, and he tells us that the varieties of the disease depend on the part of the nervous system affected; but he does not enlighten us as to the cause of this preference of the alcoholic poison. The definition of alcoholism laid down is that "when a person finds, or his friends find, that he cannot conduct himself soberly under the social conditions normal to his time, and when no persuasion, or fear, or exhortation is strong enough to induce him to reform, then he is a drunkard." But why does it happen that one man passes the boundary line and a hundred do not, and what is to be done to save the hundredth man from his self-inflicted disease, for we do not believe so much in good advice and friendly control as Dr. Wilson seems to do. For instance, take men of the army and navy, who all live under almost the same conditions. Out of the largest mess only one officer will become a drunkard, or is ever likely to become one, and the same may be said of the rank and file. Is this because the moral functions are so much perverted to begin with that the strongest possible inducements, such as loss of caste and loss of a favourite profession, fail very often to induce a cure? We have been accustomed to think that perfect recovery from the alcoholic habit was a rare occurrence; that men of that age when character is fixed seldom get beyond the emotional stage of reform; but some of the cases detailed in this work show that by hospital treatment better results may often be hoped for and sometimes obtained.

In the opening paragraphs of the section on insanity we are warned against treating the diseased brain solely from the physical standpoint. "We have forgotten," our author says, "that although it is true that a man's brain cells have become pigmented, it is an equally true statement of his insanity to say that his spirit is worn and all his feeling discoloured." Very true, but if the "spirit" here spoken of be not cerebral function, what is it? We are hand in hand with Dr. Wilson when he says that "the spiritual relation of brain cells is as big a thing and as important as their chemistry. Iron and strychnine, phosphorus and Indian hemp, are very potent remedies; but so also are fear, doubt, hope, confidence, interest, enthusiasm. Moral causes overturn many a mind and brain, and moral causes may restore them." Therefore we need not quarrel with the author, even if it be that he is at times a little more metaphysical than the philosophy of insanity warrants; and even if the "mental" physician have much difficulty in applying these "moral causes" to the cure of his insane patients. Perhaps it might be said that there is nothing very new either in the descriptions of diseases or in the therapeutics; but both drunkenness and insanity have been presented to us in a readable and entertaining form, and the treatment of both subjects is enriched with clinical details. These "cases" partake more of the character of narratives and biographical sketches than the dry excerpts from case-books hitherto so familiar to us.

THE MEDICAL ANNUAL SYNOPTICAL INDEX TO REMEDIES AND DISEASES. For the Twelve Years 1887 to 1898. (Bristol: John Wright and Co. 1899. Price 7s. 6d. net.)

THE publishers of the well-known "Medical Annual" have done well in compiling this index. Nothing is more aggravating than to be shut off from the contents of a series of volumes upon one's shelves by the necessity of consulting half a score of indices, and all who have taken the "Medical Annual" for the past few years will do well to possess themselves of this synoptical index, which gives the key to the preceding volumes. It is, however, more than a mere index. True, in some instances one is merely referred to the volume and the page where the subject required is treated of, but in the vast majority of cases the index itself contains a fair synopsis of the various points which are discussed, so that even without further reference one can in many cases gain what one wants to know from the index alone. One thing further we can say, namely, that a little examination of this index is sufficient to demonstrate to anyone the value of the Annua as a fair and representative analysis of recent medical progress.

